# Modulating microRNA Processing: Enoxacin, the Progenitor of a New Class of Drugs

**DOI:** 10.1021/acs.jmedchem.0c00510

**Published:** 2020-07-16

**Authors:** Tommaso Felicetti, Violetta Cecchetti, Giuseppe Manfroni

**Affiliations:** Department of Pharmaceutical Sciences, University of Perugia, via del Liceo 1, 06123 Perugia, Italy

## Abstract

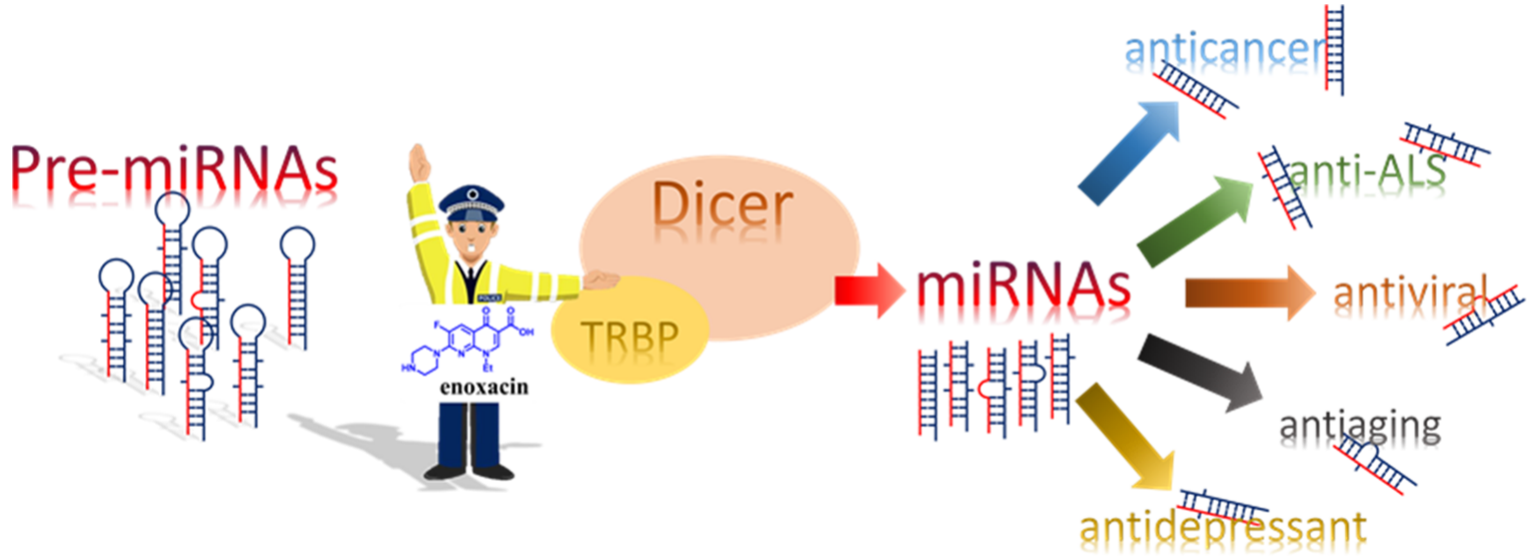

The RNA interference (RNAi) process encompasses the cellular mechanisms by which short-noncoding RNAs posttranscriptionally modulate gene expression. First discovered in 1998, today RNAi represents the foundation underlying complex biological mechanisms that are dysregulated in many diseases. MicroRNAs are effector molecules of gene silencing in RNAi, and their modulation can lead to a wide response in cells. Enoxacin was reported as the first and unique small-molecule enhancer of microRNA (SMER) maturation. Herein, the biological activity of enoxacin as SMER is discussed to shed light on its innovative mode of action, its potential in treating different diseases, and the feasibility of using enoxacin as a chemical template for inspiring medicinal chemists. We debate its mechanism of action at the molecular level and the possible impact on future ligand and/or structure-guided chemical optimizations, as well as opportunities and drawbacks associated with the development of quinolones such as SMERs.

## Overview of RNAi

Until some years ago, most scientific studies had been directed toward the understanding of protein-coding DNA regions, thus ignoring the remaining DNA considered by many as “junk”. Much has been made of the year 1993, when two independent studies led to the discovery that a short noncoding region of DNA (*lin-4*) was involved in negatively regulating the expression of LIN-14 in *Caenorhabditis elegans*.^1,2^ A few years later (1998), Andrew Fire and Craig Mello published their breakthrough study on the mechanism of RNA interference (RNAi)^3^ and were subsequently awarded the Nobel Prize in Physiology or Medicine in 2006.^4^

**Figure 1:**
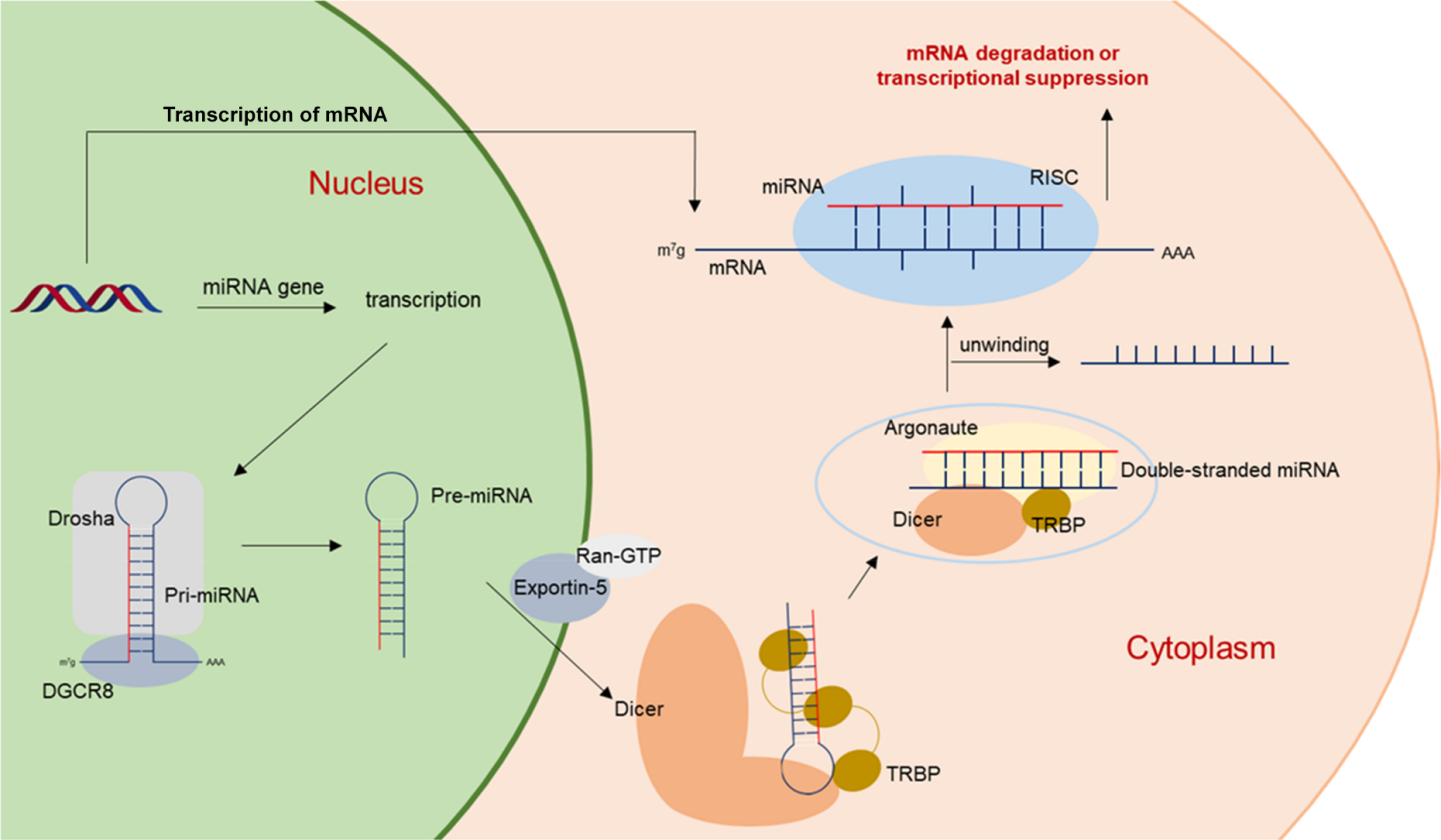
miRNA biogenesis pathway.

Today, we know that RNAi is a posttranscriptional process triggered by double-stranded RNA (dsRNA), which leads to gene silencing in a sequence-specific manner. MicroRNAs (miRNAs), small noncoding RNAs constituting approximately 22 nucleotides, play a key role in RNAi. By targeting a specific mRNA through base pairing, miRNAs lead to its degradation or translational suppression. To date, more than 1,000 miRNAs have been discovered and more than 60% of human protein-coding genes contain at least one conserved miRNA-binding site.^5^ The miRNA biogenesis (outlined in Figure 1) begins in the nucleus after RNA-polymerase II-mediated transcription to generate a primary transcript (pri-miRNA) that in turn is cleaved by the RNase III Drosha in combination with the double-stranded RNA (dsRNA) binding protein (dsRBP) named DGCR8 to a shorter hairpin precursor having approximately 70 nucleotides (pre-miRNA). After export to the cytosol by Ran-GTP/Exportin-5, the RNase III Dicer cleaves the loop of pre-miRNA to generate a mature double-stranded miRNA by interacting with the cofactor TAR-RNA binding protein (TRBP), a double-stranded RNA binding protein that was first identified as a cellular protein that facilitates the replication of human immunodeficiency virus.^6^ Subsequently, the Dicer/TRBP/miRNA complex is loaded onto Argonaute proteins that, after duplex miRNA unwinding, generate the RNA-induced silencing complex (RISC) including the single-stranded miRNA responsible for specific mRNA silencing (degradation/suppression).^5^ The seed region of mature miRNAs is typically a sequence of 6–8 nucleotides at the 5′ end with partial or total complementarity to the 3′ untranslated region (UTR) of the mRNA target.^7^ Each miRNA has hundreds of target mRNAs, and miRNAs having a common seed region regulate the same targets.^8^ In this manner, miRNAs play a crucial role in various processes such as tissue development, morphogenesis, apoptosis, and signal transduction pathways, thus showing involvement in both physiological pathways and numerous diseases.^9,10^

Tuning miRNA pathways has attracted great interest in the past decade, leading to different strategies aimed at targeting specific miRNAs or mRNAs involved in various diseases. miRNA and/or short-interfering RNA (siRNA)-based therapeutics have been developed, culminating in tens of molecules facing clinical trials.^11^ Patisiran, the first siRNA-based drug approved by the FDA in 2018 to treat polyneuropathy, sheds light on the high potential of this strategy.^12^ However, the downside, represented by ADME properties, still poses major limitations and tough challenges for the development of these innovative RNA-based therapeutics. The identification and design of accurate and specific delivery systems for each miRNA/siRNA drug is challenging but essential to enable tissue-specific targeting and reduce toxicity and off-target effects, thus extending the development time for these drugs. In addition, this kind of drugs suffers from poor stability in gastric fluids as well as low intestinal adsorption, thereby being administrable only for intravenous and subcutaneous rather than oral administration.^11^

In recent years, a part of the research in the RNAi field has been focused on the identification of small molecules that inhibit the expression of specific miRNAs, named small molecule inhibitors of specific miRNAs (SMIRs).^13^ The first example of a SMIR reported in the literature appeared in 2008 as a result of a screening performed on 1,000 compounds followed by medicinal chemistry optimization.^14^ The first identified SMIR, a diaminohexahydropyrimidine derivative, was able to strongly decrease miR-21 expression endogenously expressed in different cancer cell lines. However, insights into the mechanism of action showed that the SMIR targeted the transcription of miR-21 gene into pri-miR-21 but not downstream processes of the common miRNA pathway.^14^ Since then, several research groups initiated studies focused on the identification of new SMIRs,^15^ but further details are beyond the scope of this review.

**Figure 2:**
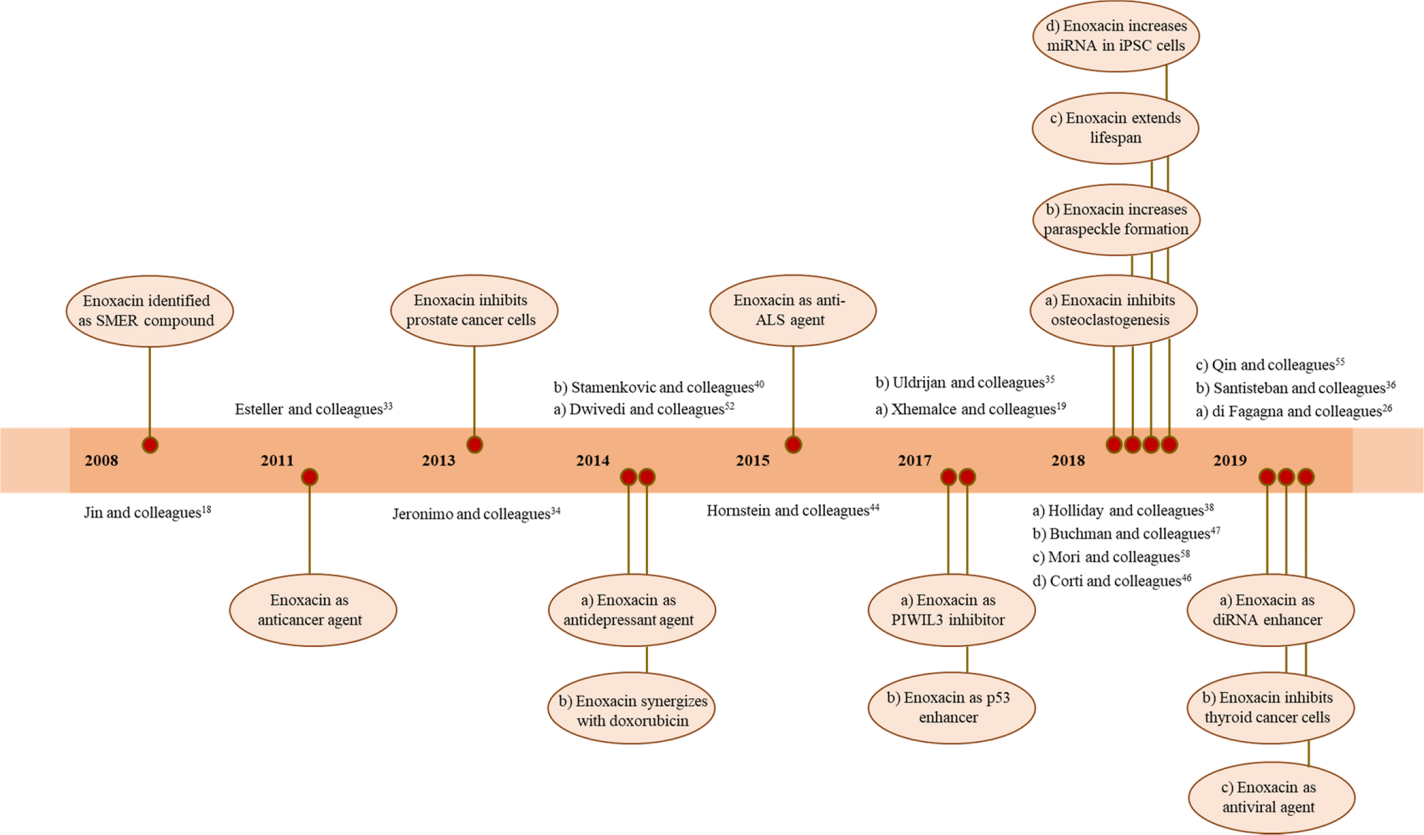
Timeline reporting the findings for enoxacin as a SMER compound over the years.

Although different approaches have been pursued to reduce the expression of specific miRNAs, new findings suggest that miRNA expression is widely suppressed in different diseases, thus focusing attention toward new strategies aimed at stimulating miRNA biogenesis.^16,17^ Nonetheless, research toward the identification of a small-molecule enhancer of microRNA (SMER) maturation did not attract great attention until the appearance of enoxacin, the sole SMER reported in the literature to date. In this review, insights on its discovery and associated research are described. Potential applications as well as future directions for the development of SMER compounds are discussed with the aim to boost scientific research in this field, especially from a medicinal chemistry stance. In this review, we illustrate the milestones that led enoxacin to becoming a validated chemical probe, which is now used in biological processes involving the modulation of RNAi. The logical thread we follow does not exactly correspond to the timeline of the discoveries but considers the findings in sections based on activities. However, the timeline of papers appearing in the literature regarding enoxacin as a SMER is reported in Figure 2 and Table 1.

**Table 1:** Different Activities of Enoxacin as a SMER Compound Reported in the Literature

Activity	Work highlights	Year of publication	Reference
RNAi and miRNA maturation enhancer	• Identification of enoxacin as a miRNA enhancer from screening of 2,000 US FDA drugs.	2008	Jin et al.^a^
	• Experimental evidence of the involvement of TRBP in the SMER activity of enoxacin.		
Anticancer agent	• Identification of enoxacin as an anticancer agent in a panel of 12 cancer cell lines.	2011	Esteller et al.^b^
	• Confirmation of its TRBP-dependent activity and evidence of TRBP/enoxacin binding by biophysical experiments.		
Anticancer agent	• Identification of the ability of enoxacin to inhibit the growth of prostate cancer cells through the reduction of HDAC1 and SIRT1 protein levels by miRNA modulation.	2013	Jeronimo et al.^c^
Anticancer agent	• Identification of a synergistic activity between enoxacin and doxorubicin in treating Ewing sarcoma family tumors.	2014	Stamenkovic et al.^d^
Anticancer agent	• Identification of the ability of enoxacin to inhibit the growth of melanoma cell lines.	2017	Uldrijan et al.^e^
	• Experimental evidence that enoxacin increases p53 protein levels by reducing MdmX transcripts through miRNA modulation.		
	• Demonstration that enoxacin is not involved in human topoisomerase inhibition.		
Anticancer agent	• Demonstration of the ability of enoxacin to inhibit osteoclast formation by miRNA modulation.	2018	Holliday et al.^f^
Anticancer agent	• Identification of the ability of enoxacin to inhibit the growth of a human thyroid cancer in an orthotopic mouse model.	2019	Santisteban et al.^g^
	• Experimental evidence of the ability of enoxacin to restore miRNA maturation at physiological levels in cells with low levels of Dicer.		
Anticancer agent	• Identification of the ability of enoxacin to stimulate diRNA production promoting DNA repair.	2019	D’Adda di Fagagna et al.^h^
Anti-ALS agent	• Identification of enoxacin as an anti-ALS agent by its ability to modulate miRNA maturation.	2015	Hornstein et al.^i^
	• Demonstration that enoxacin delays neurological symptoms in ALS mice.		
Anti-ALS agent	• Identification of the ability of enoxacin to increase miRNA maturation in induced pluripotent stem cells (iPSCs).	2018	Corti et al.^j^
Anti-ALS agent	• Identification of the ability of enoxacin to increase paraspeckle formation in neuroblastoma cells.	2018	Buchman et al.^k^
Antidepressant agent	• Identification of the ability of enoxacin to reduce depressive behavior in rats by miRNA modulation.	2014	Dwivedi et al.^l^
Antiviral agent	• Identification of the ability of enoxacin to prevent Zika virus infection in hNPC cells.	2019	Qin et al.^m^
	• Experimental evidence that the antiviral activity of enoxacin is dependent on the presence of Dicer.		
Antiaging agent	• Identification of the ability of enoxacin to increase lifespan in *C. elegans* by modulating miRNA maturation.	2018	Mori et al.^n^
	• Experimental evidence that lifespan-increasing activity of enoxacin is ADAR-dependent.		

a Reference (18).

b Reference (33).

c Reference (34).

d Reference (40).

e Reference (35).

f Reference (38).

g Reference (36).

h Reference (26).

i Reference (44).

j Reference (46).

k Reference (47).

l Reference (52).

m Reference (55).

n Reference (58).

## Enoxacin: The First and Sole SMER Reported in the Literature to Date

**Figure 3:**
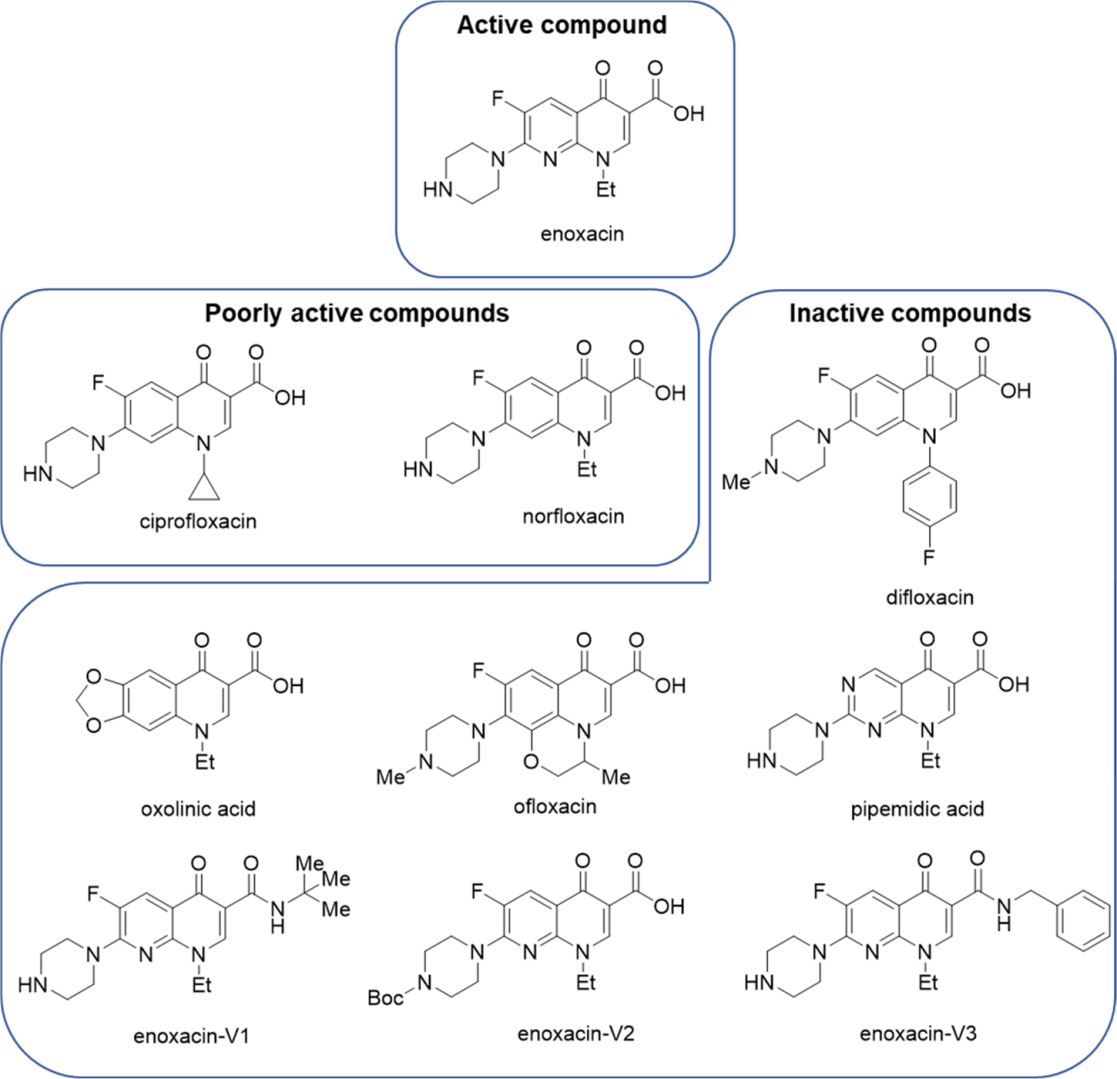
Enoxacin and analogues tested by Jin and colleagues as RNAi modulators. Among the assayed quinolones, only enoxacin exerted noteworthy activity.

In 2008, by screening of 2,000 US Food and Drug Administration-approved compounds and natural products, Jin et al. at Emory University reported, for the first time, the small-molecule enoxacin as an RNAi enhancer (Figure 2, Table 1).^18^ Enoxacin (Figure 3) is an oral broad-spectrum fluoroquinolone bactericidal agent that inhibits DNA gyrase and topoisomerase IV but is unable to interfere with human topoisomerases. Enoxacin was identified as an RNAi enhancer via a reporter assay performed with 2,000 molecules using human embryonic kidney (HEK293) cells expressing the gene encoding 293-EGFP (enhanced green fluorescent protein) infected with a lentivirus expressing a short-hairpin RNA (shRNA). By the RNAi mechanism, shRNA is processed in siRNA that specifically targets the mRNA transcripts of the 293-EGFP, thereby reducing their translation. Compounds that are able to enhance the RNAi mechanism have been expected to increase siRNA formation and in turn reduce EGFP-mediated fluorescence. Of 2,000 compounds, only enoxacin reduced fluorescence, showing a dose-dependent effect (EC_50_ ≈ 30 μM). In addition, enoxacin lost its activity when the assay was repeated in the absence of shRNA, thus showing its role in increasing siRNA production. In parallel, experiments in the presence of different shRNAs, specifically designed to reduce the expression of a variety of proteins (i.e., luciferase and Fmr1), were also carried out; enoxacin retained its ability to enhance siRNA production, thereby highlighting a universal effect that was not only dependent on the siRNA targeting the 293-EGFP mRNA. Unexpectedly, the RNAi enhancing effect of enoxacin appeared to be structure-dependent since other related compounds belonging to the same fluoroquinolone class did not possess this ability (Figure 3). Indeed, when setting the RNAi-enhancing activity of enoxacin as 100%, only ciprofloxacin and norfloxacin exhibited an activity greater than 50%. Although the authors did not comment on any type of structure–activity relationship (SAR), we extrapolated some useful clues: (*i*) carboxylic function as well as the free piperazine nitrogen are needed to retain the RNAi-enhancing effect (both features are present in enoxacin, norfloxacin, and ciprofloxacin); (*ii*) whether the carboxylic function and piperazine moiety appear essential, they are not sufficient to obtain the RNAi-enhancing activity, as demonstrated by pipemidic acid, which, in contrast to enoxacin, norfloxacin, and ciprofloxacin, possesses a pyridopyrimidine backbone; (*iii*) focusing attention on the molecule backbone, the 1,8-naphthyridone moiety seems to be preferred over the quinolone scaffold, as demonstrated by the comparison between enoxacin and norfloxacin, which differ only in position 8 for the presence of a nitrogen. Nonetheless, given the small number of reported fluoroquinolones, a robust SAR analysis is not possible.

In the same work focusing attention on the miRNA maturation pathway, the authors observed an increase in miR-125a in HEK293 cells treated with enoxacin and stably expressing pri-miR-125a transcripts, suggesting that enoxacin can promote the processing of miR-125a and, thus, exhibit its SMER effect. Moreover, when performing miRNA TaqMan assays to monitor the profiles of 157 miRNAs in HEK293 cells treated with enoxacin, only 15 out of 157 endogenous miRNAs were significantly affected (i.e., let-7b, miR-124a, miR-125a, miR-139, miR-146, miR-152, miR-190, miR-199a*, miR-199b, miR-23a, miR-30a, miR-96, miR-99a, let-7i, and miR-128b). In contrast to all miRNAs, in a parallel experiment on untreated HEK293 cells, the authors observed that the increase in miRNAs was associated with high levels of its own precursors, thereby suggesting that enoxacin could promote Dicer processing activity without influencing miRNA precursor expression.

In further experiments, enoxacin showed no effects on the maturation of pre-let-7 and pre-miR-30a by *in vitro* Dicer-mediated processing assays (in the absence of TRBP). In contrast, when processing experiments were repeated in the presence of the cofactor TRBP, enoxacin significantly enhanced miRNA maturation differently from oxolinic acid used as a negative control. Therefore, as stated by the authors, enoxacin activity was TRBP-dependent and likely involved improvement of TRBP-pre-miRNA affinity, as also shown by binding assays displaying a decrease in the K_D_ between TRBP and pre-miRNA in the presence of enoxacin (from 221 nM to 94 nM). Of note, the RNAi enhancing activity mediated by enoxacin was also confirmed in *in vivo* studies performed using GFP transgenic mouse models injected with a lentivirus expressing shGFP.

Regarding the molecular target recognized by enoxacin, an additional protein has been proposed that merits mentioning. In fact, in 2017 (Figure 2),^19^ in an attempt to directly identify the molecular target of enoxacin, Xhemalce and colleagues performed a pull-down experiment with streptavidin beads using a close derivative of enoxacin that was directly reacted by click chemistry in the lysate of MCF7 cells. Surprisingly, analysis of the experiment by SDS-PAGE and high-resolution mass spectrometry revealed PIWIL3 as the potential target. Data authenticity regarding PIWIL3 was confirmed by Western blot analysis using an anti-PIWIL3 antibody. PIWIL3 belongs to the PIWI argonaute proteins involved in the maturation of the Piwi-interacting RNAs (piRNAs), small noncoding RNAs that differ from miRNAs.^20^ Although mostly present in normal testis tissue, PIWIL3 has been reported to be aberrantly expressed in a variety of cancers, playing important roles in tumorigenesis.^21,22^ When the authors depleted PIWIL3 in MCF7 cells, increased miRNA levels and growth defects were detected, similar to the behavior observed for MCF7 cells treated with enoxacin. Taken together, these results strongly suggested an additional molecular target for enoxacin, necessitating further detailed investigations. A comparison between the targets identified for enoxacin will be the objective of a specific section titled Considerations of the Mechanism of Action in the Context of Drug Discovery.

## Enoxacin, Not Only an RNAi Enhancer

The recent findings that small noncoding RNAs, specifically DNA double-strand break-induced RNAs (diRNAs), appeared to be involved in DNA repair, laid the foundation for a broader view of the RNAi machinery, thus overcoming the specific role in gene silencing toward the more complex mechanism of DNA-damage response (DDR) modulation.^23,24^ The concomitant discovery that Dicer accumulated at DNA double-strand break sites to catalyze diRNA formation^25^ led d’Adda di Fagagna et al. (Figure 2, Table 1), in 2019, to evaluate the ability of enoxacin to stimulate DNA-damage response RNA (DDRNA) maturation.^26^ HeLa cells treated with enoxacin (50 μM) before exposure to ionizing radiation exhibited increased DDR activation with respect to control cells treated with DMSO. The authors specified that enoxacin did not directly increase the expression of the studied proteins involved in DDR (γH2AX, pATM^S1981^, 53BP1, MDC1, and pS/TQ) but strongly boosted their activation. In addition, the authors observed that enoxacin led to an increase in the levels of the phosphorylated forms of pCHK2^T68^ and p53^S15^, both effector kinases and direct substrates of the Ataxia Telangiectasia Mutated (ATM) kinases, thus highlighting the ability of enoxacin to mediate the ATM-CHK2-p53 signaling pathway.

To verify whether enoxacin-mediated diRNA maturation was TRBP-dependent, in the same work, the authors investigated the activity of enoxacin in HeLa cells in which TRBP was knocked down. In accordance with the miRNA-enhancing maturation, the absence of TRBP in HeLa cells completely prevented enoxacin-mediated DDR activation.

In addition, when the authors monitored DDRNA expression, an event mediated by Dicer activity, in NIH2/4 cells (wt TRBP) by qRT-PCR analysis, enoxacin was directly able to enhance it in a manner similar to the increase observed for miR-29b, a miRNA involved in enoxacin-mediated maturation. Thus, enoxacin once again displayed that its activity was dependent on TRBP; indeed, as confirmation, its activity was abrogated by TRBP depletion.

To understand how enoxacin promoted DDR, in the same paper, the authors investigated the two known DNA repair mechanisms, homologous recombination (HR) and nonhomologous end joining (NHEJ), upon enoxacin treatment in the U2OS cell line. Interestingly, enoxacin significantly improved the NHEJ mechanism, in turn reducing HR efficiency. Since NHEJ is known as an error-prone repair mechanism,^27^ the authors also evaluated the ability of enoxacin to refine the accuracy of the NHEJ mechanism and, surprisingly, observed that enoxacin positively impacted NHEJ accuracy by promoting accurate DNA repair.

## The SMER Enoxacin as an Anticancer Agent

Cancer represents one of the most serious diseases in which genetic alterations lead to dysregulation of the wide and fine machinery making up the complex cellular signaling pathway.

In the cancer field, miRNAs can be grouped in two different classes: those targeting tumor suppressor proteins, also called tumor suppressive miRNAs, and those targeting oncogenes.^28^ Dysregulation of miRNAs underlies the transformation of normal cells into malignant ones. Focusing attention on the miRNA pathway, two different anticancer strategies can be applied: (*i*) repression of miRNA function to prevent mRNA silencing of onco-suppressor genes or (*ii*) enhancing of miRNA function to degrade mRNA encoded by oncogenes.

To date, the former approach relies on antisense oligonucleotides (antimiRs), locked nucleic acids (LNAs), LNA-antimiR constructs, antagomirs, miRNA sponges, ribozymes/DNAzymes, siRNAs, and shRNAs.^13^ Although all these molecules represent an exciting strategy in counteracting cancer, as previously stated for miRNA/siRNA-based therapeutics, some issues remain to be fixed; the delivery system and pharmacodynamic and pharmacokinetic properties pose a hard challenge to overcome before therapeutic application.^15,29,30^ All these approaches can be grouped in the first set of therapeutics concerning the repression of miRNA function.

Conversely, to date, little is known about molecules belonging to the second set, which are able to enhance miRNA function to degrade mRNA encoded by oncogenes. These molecules show potential in cancer therapy, especially considering some recent findings where the expression levels of miRNA-processing machinery components (i.e., Drosha and Dicer) were decreased in some cancers, which was often associated with a poor clinical outcome.^31,32^ In addition, there have been reports showing that miRNA biogenesis machinery components exhibit normal expression in cancer, but with a widespread suppression of miRNAs.^16^

In 2011, Esteller and colleagues (Figure 2, Table 1) described the effect of enoxacin on a panel of 12 cancer cell lines after 5 days of exposure, and the EC_50_ (40 μg/mL or 124 μM) was evaluated using the HCT-116 cell line.^33^ Enoxacin reduced cell viability in all cancer cell lines while not affecting primary cells, thus displaying a cancer-specific growth-suppressive activity. Based on the previous results reporting that enoxacin could enhance miRNA processing,^18^ the authors monitored the maturation of specific tumor-suppressor miRNAs in two enoxacin-treated cancer cell lines (HCT-116 and RKO). In that experiment, enoxacin increased the production of 24 mature miRNAs and in turn reduced the amount of the corresponding precursor pre-miRNAs.^33^ According to the previous findings of Jin et al., among the affected miRNAs, the authors identified let-7 and miR-125a. In a wider analysis of the whole miRNA expression profile, it was evident that enoxacin was able to enhance miRNA biogenesis, upregulating 100 out of 122 differently expressed miRNAs. Interestingly, most of these upregulated miRNAs could be associated with a tumor-suppressor effect.

To evaluate whether enoxacin could enhance miRNA processing by binding to TRBP, in the same work, biophysical experiments were performed using surface plasmon resonance (SPR) and isothermal titration calorimetry against wild-type and mutated TRBP proteins. Of note, enoxacin was able to bind in the low micromolar range to wild-type TRBP but not the mutated TRBP. In addition to the biophysical assays, a TRBP-dependent effect of enoxacin was also demonstrated in *in vitro* assays using human cells carrying TRBP mutants. In three tumor human cell lines having different mutations of TRBP, enoxacin did not reduce cell viability, thus confirming its TRBP-dependent mechanism of action. This trend was also retained in *in vivo* animal tumor models using both xenografted cancer cells and primary colorectal tumors. Most importantly, enoxacin did not show any toxic effect at the dose used (10 mg/kg i.p. daily injection for 4 weeks).

Following this pioneering research, in subsequent years, a series of studies have been published examining the antitumor effect of enoxacin toward specific human cancers. Jeronimo et al. (Figure 2, Table 1) in 2013 studied the ability of enoxacin in the growth inhibition of different prostate cancer cells (LNCaP and DU145)^34^ with an EC_50_ of 105 μM and 141 μM, respectively. Thus, the authors performed an extensive analysis of the miRNA expression profile in a panel of 742 miRNAs upon enoxacin treatment. Enoxacin was able to differentially modulate the expression of 122 and 147 miRNAs in LNCaP and DU145 cells, respectively. Upregulation occurred in 53% of cases (65 of 122) in LNCaP cells and 60% (88 of 147) in DU145 cells. Focusing their attention on specific miRNAs involved in prostate cancer, in the same work, the authors observed a significant increase in the biogenesis of the tumor-suppressor miR-17*, miR-29b, miR-34a, miR-132, miR-146a, and miR-449a coupled with a reduction of the oncogenic miR-141 and miR-191 after enoxacin treatment. Since HDAC1 and SIRT1 proteins are targeted by miR-449a and miR-34a, respectively, their levels were measured by Western blotting to indirectly estimate the enhancement of miRNA maturation. According to the hypothesis, HDAC1 and SIRT1 protein levels decreased after enoxacin exposure in both cell lines.

A few years later, Uldrijan et al. (Figure 2, Table 1) demonstrated that enoxacin at 156 μM also displayed antitumor effects on human melanoma cell lines during a five-day treatment.^35^ Enoxacin was effective regardless of common mutations in melanoma cells such as on BRAF kinase or NRAS oncogene. In the same article, by using A375 cells with enoxacin, the authors performed an Affymetrix GeneChip miRNA assay containing 5706 probe sets and observed that it was able to modify the expression levels of 55 maturated miRNAs, in particular upregulating 26 of them. After a thorough analysis concerning the potential targets of the affected miRNAs, the authors focused attention on miR-3154 and miR-4459, which were predicted as the target of the human MdmX (*Mdm4*) transcript. The potential interference with MdmX expression can strongly impact p53 protein levels and thus significantly influence apoptotic cell pathways. Indeed, after a 24-h incubation with A375 cells, enoxacin (25 μg/mL) increased p53 protein levels simultaneously producing a slight decrease in MdmX levels, mostly when enoxacin was used at higher doses (75 and 100 μg/mL). To validate that the antitumor effect of enoxacin involved the increase in p53 levels, different p53 targets such as *BBC3/PUMA*, *p21/CDKN1A*, *GADD45*, and *MDM2* were monitored and found to be effectively reduced in A375 cells. In the same work, the authors observed that ciprofloxacin (Figure 3), in contrast to ofloxacin (Figure 3), was able to reproduce, although to a lesser extent, a comparable profile to enoxacin; this finding is in agreement with previous studies by Jin and colleagues.^18^ However, the authors wondered whether enoxacin could enhance the p53 response by acting as a DNA-damaging agent (never excluded previously), similar to doxorubicin or etoposide. When monitoring the stabilization of p53 protein after 3 and 6 h, there was a significant increase in p53 stabilization in A375 cells treated with doxorubicin and etoposide, while no response was observed in the presence of enoxacin. In addition, a strong phosphorylation of p53 and histone H2AX was observed only in cells treated with DNA-damaging agents. Enoxacin did not induce such a phosphorylation, thus indirectly validating that its antitumor activity was DNA damage-independent.

Attention focused on enoxacin has increased over the years, and in 2019, Santisteban et al. (Figure 2, Table 1) observed that in thyroid cancer cells, miRNAs differing from the oncogenic miR-146b-5p (e.g., miR-146b-3p, miR-221-3p, miR-222-3p, miR-21-5p, miR-21-3p and miR-182-5p) were overexpressed and involved in reducing Dicer expression.^36^ Therefore, all of them potentially act as negative feedback regulators of Dicer expression, in turn producing a global downregulation of miRNAs. Among these downregulated miRNAs, the authors observed a downregulation of some tumor suppressor miRNAs such as miR-30a-5p, miR-30a-3p, miR-100, and miR-204.^36^ Interestingly, in the same work, the authors also observed that a similar profile of miRNA downregulation could be obtained when silencing Dicer in Cellosaurus Cal62 and TPC1 cells, thus producing an increase in terms of migration and invasion, and in protein markers involved in epithelial-mesenchymal transition (EMT). Based on these findings, the authors rationalized that enoxacin could reverse the effect of the reduced Dicer levels. Thus, Cal62, TPC1, and SW1736 cells, overexpressing miR-146b-5p, were treated with enoxacin (40 μg/mL) and showed a significant decrease in terms of proliferation, migration, and EMT markers, similarly to Dicer-silenced cells treated with miR-30a and miR-100. This antitumor effect was further confirmed *in vivo* using an orthotopic mouse model of human thyroid cancer treated with 15 mg/kg of enoxacin for 28 days. We want to highlight that in addition to the effect of enoxacin in inhibiting cancer cell growth, several studies demonstrated, for the first time, the relevance of restoring/enhancing global miRNA expression in the treatment of cancer.

Focusing attention toward bone cancer, enoxacin has previously been reported as a small molecule that is able to inhibit osteoclast formation by indirectly blocking the interaction between vacuolar H^+^-ATPase and microfilaments.^37^ However, the mechanism of action underlying this effect is still poorly understood. Recently, based on the aforementioned results, Holliday et al. (Figure 2, Table 1) observed that the enoxacin-mediated osteoclastogenesis inhibition could be related to the ability of enoxacin at 50 μM to increase levels of miR-214-3p.^38^ However, concentrations of enoxacin higher than 100 μM were required for growth inhibition of 4T1 cancer cells, the murine breast cancer cells commonly used to study the ability of breast cancer to invade bone. Further investigations showed that 4T1 cancer cells treated with enoxacin at 50 μM generated significantly smaller extracellular vesicles (EVs) than the control. EVs have emerged as important intercellular regulators in cancer invasion and in regulating bone remodeling.^39^ Therefore, Holliday et al. observed that, after treatment with enoxacin, 4T1-derived EVs showed higher levels of miR-214-3p and lower levels of miR-146a-5p and let-7b-5p than the control. When these EVs were used to treat calcitriol-stimulated mouse marrow, strong osteoclast formation inhibition was observed compared with the control.^38^ Therefore, enoxacin was able to exert two different effects depending on the concentration used (inhibition of osteoclastogenesis at 50 μM and of cancer cell proliferation at >100 μM); however, both effects appeared to be related to the modulation of miRNA biogenesis.

The anticancer effect of enoxacin was also recently reported by d’Adda di Fagagna and colleagues (Figure 2, Table 1) in the research article in which enoxacin was identified as a potential diRNA enhancer (see above for details).^26^ The authors presented a relevant point. A molecule that is able to repair DNA damage could counteract the anticancer activity of DNA-damaging agents such as etoposide or doxorubicin. Indeed, when the authors incubated U2OS and HeLa cells with increasing amounts of etoposide or doxorubicin in the presence of enoxacin, they observed a significant increase in the IC_50_ of the chemotherapeutics, thereby confirming their concerns regarding a potential interference between DNA-damaging agents and enoxacin (or similar agents) for future anticancer therapy.

The warning provided by d’Adda di Fagagna and colleagues that enoxacin cannot be used in combination with DNA-damaging agents should be carefully considered in each individual case. In our opinion, the combinations between enoxacin and DNA-damaging agents must be studied and understood in greater detail, especially in view of the opposite finding previously obtained by Stamenkovic and colleagues.^40^ Indeed, the authors observed a synergistic activity between enoxacin and doxorubicin in treating Ewing sarcoma family tumors (EFST) both in *in vitro* and *in vivo*. In such synergism, the ability of enoxacin relies on inhibiting, at mild concentrations (those used for the antibacterial activity), cancer stem cell growth, which is only moderately inhibited by doxorubicin alone. In contrast, doxorubicin was able to strongly deplete the rapidly dividing cells that formed the tumor mass of the EFST.

## Enoxacin: A New Hope against Amyotrophic Lateral Sclerosis

We have dedicated a large section to the effects of enoxacin on tumors, but another very interesting application is the potential treatment of amyotrophic lateral sclerosis (ALS). ALS belongs to a wider group of disorders known as motor neuron diseases, which are caused by the degeneration and death of motor neurons. Extending from the brain to the spinal cord and to muscles throughout the body, motor neurons are nerve cells that initiate and provide vital communication links between the brain and voluntary muscles. However, in ALS, both the upper and lower motor neurons degenerate or die and stop sending messages to the muscles. Unable to exert their function, the muscles gradually weaken, start to twitch (fasciculations), and waste away (atrophy).^41^ Currently, there is no effective cure for ALS and no effective treatment to stop, or reverse, the progression of the disease.^41^

Stress and stress granules (SGs), cytoplasmic sites for modulating mRNA translation, could be involved in the pathogenesis of ALS.^42^ Dicer and its cofactors have been reported to participate in the mechanisms involved in the stress response;^42^ Dicer deficiency reduces stress tolerance,^43^ and the consequent loss of miRNA biogenesis causes spinal motor neuron degeneration.^17^ Based on these findings, in 2015, Hornstein and colleagues published a paper (Figure 2, Table 1) in which enoxacin was used as a chemical probe to modulate miRNA processing in NSC-34 cells carrying mutant forms of proteins responsible for the ALS phenotype (FUS, TDP-43, and SOD1).^44^ Indeed, cells carrying mutant proteins showed decreased expression of miRNAs despite retaining a normal Dicer level. When enoxacin (100 μM) was administered to mutated NSC-34 cells, miRNA maturation was improved after 72 h by restoring a miRNA expression profile comparable to wild-type cells. Of note, this effect was retained in a mouse model of ALS (SOD1 mutation) in which enoxacin enhanced several miRNAs in motor cortices. Enoxacin displayed the ability to delay neurological symptoms by 7 days in ALS mice (SOD1 mutation) when compared with controls.

This enoxacin-mediated activity observed in the ALS models appeared to be similar to that observed in thyroid cancer cells in which Dicer activity was negatively regulated by miR-146b-5p.^45^ Thus, although the diseases are widely different, the increase in miRNA maturation and processing up to physiological levels appear to be a common route to fight both cancer and ALS. Thus, enoxacin appears to improve miRNA maturation in a context where Dicer struggles to perform its dicing activity.

In the wake of this finding, in 2018, Corti et al. investigated the role of enoxacin in induced pluripotent stem cells (iPSCs).^46^ They reprogrammed fibroblasts from sporadic and familial ALS patients into iPSCs and evaluated miRNA expression profiles of motor neuron progenitors. With respect to the control, motor neuron progenitors exhibited decreased expression of 15 miRNAs, in particular, miR-504, miR-429, miR-34a, miR-133a, miR-7-2*, and miR-1225-3p. To identify potential targets linked to these dysregulated miRNAs, the authors performed a bioinformatics analysis. By reducing the choice of targets involved in the central nervous system, the authors identified miR-34a and miR-504 as miRNAs likely having a key role in ALS pathology. Considering the role of enoxacin in improving dicing activity, the authors investigated the expression of miR-34a and miR-504 in iPSC cells treated with enoxacin (100 μM) for 48 h and observed a significant increase in both miRNAs with respect to the control treatment group.

In the same year, Buchman et al. (Figure 2, Table 1) showed a further effect of enoxacin in ALS.^47^ They observed that in SH-SY5Y neuroblastoma and MCF7 cells, defects in the miRNA biogenesis machinery led to an increase in the formation of paraspeckles, which are subnuclear bodies localized in mammalian cells that control gene expression.^48^ In this specific case, depletion of TAR DNA-binding protein 43 (TDP-43) in cells increased paraspeckle formation, according to the role of TDP-43 in contributing to miRNA biogenesis.^49^ It is known that paraspeckles can compensate for TDP-43 depletion by spatially organizing proteins involved in miRNA biogenesis.^50^ When Buchman et al. treated neuroblastoma cells with enoxacin (10 μM), paraspeckle assembly was increased after 24 h.^47^ Such an effect could occur as a result of an increase in miRNA biogenesis, thus again confirming the indirect but key role of enoxacin in increasing miRNA maturation in ALS.

As a consequence of these studies, enoxacin received an orphan designation by the European Medicines Agency (EMA) for ALS treatment in 2015 (EU/3/15/1459); to date, no further information regarding the outcomes of the study is available.^51^

## Enoxacin-Mediated miRNA Modulation Leaves Room for a Wide Panel of Applications

Until now, we have described the effects of enoxacin on cancer and, to a lesser extent, ALS, focusing attention on the peculiar mechanism of action aimed at modulating miRNA processing by Dicer. However, due to its effect on this complex process, the activity of enoxacin can be extended to many other applications.

In 2014, Dwivedi et al. (Figure 2, Table 1) reported the ability of enoxacin to enhance miRNA expression in the rat frontal cortex.^52^ Based on previous discoveries showing that (*i*) downregulation of miRNAs is present in depressed suicide subjects and (*ii*) rats exposed to repeated inescapable shock exhibit differential miRNA changes depending on whether they present normal adaptive responses or learned helpless behavior,^53^ the authors investigated the role of enoxacin in modulating the rat response to inescapable shock.^52^ Of course, enoxacin was selected considering its ability to interfere with miRNA maturation and its known capability to cross the blood–brain barrier, given its use as an antibacterial to treat meningitis.^54^ When intraperitoneally administered to rats, enoxacin (10 mg/kg) increased miRNA levels by 3- to 12-fold, especially considering specific miRNAs involved in neuronal cell biology such as let-7a, miR-124, miR-125a-5p, and miR-132. Pretreated rats with enoxacin showed a reduced learned helpless behavior when subjected to inescapable shock. We underline that this represents the first example of a small molecule capable of reducing a depressive behavior by modulating miRNA processing.

Considering the acquired fame in modulating miRNA processing, enoxacin has recently been employed by Qin and colleagues as an antiviral agent that is able to prevent Zika virus (ZIKV) infection by interfering with miRNA expression in human neural progenitor cells (hNPCs).^55^ ZIKV is a positive-sense, single-stranded RNA virus belonging to the genus *Flavivirus*; it spreads mostly by the bite of an infected *Aedes* species mosquito (*Ae. aegypti* and *Ae. albopictus*). Zika infection during pregnancy can cause a birth defect of the brain called microcephaly and other severe brain defects.^56^ hNPCs, which give rise to the building blocks of the human cortex, are the major target cells of ZIKV, which damages their proliferation and differentiation and causes extensive cell death.^57^ In their manuscript,^55^ Qin et al. (Figure 2, Table 1) reported that, unlike more differentiated cells, hNPCs lacked the IFN-based immune response to counteract ZIKV, and the main response to ZIKV of hNPC cells consisted of an antiviral RNAi mechanism. However, through Dicer, viral RNAs were transformed in virus-derived small interfering RNAs (vsiRNAs) that are loaded on the cellular RISC to cleave cognate viral RNAs, but the downregulation of Dicer in infected hNPC cells enhanced ZIKV replication. Enoxacin (100 μM) prevented ZIKV infection in hNPC cells, thus suggesting that its known ability to modulate miRNA processing and RNAi mechanisms could prevent viral infections in hNPC cells. Enoxacin showed an IC_50_ of 51.99 μM and a CC_50_ of 175.8 μM. In contrast, in knockout Dicer hNPC cells, enoxacin failed to prevent viral infection, suggesting its involvement in RNAi mechanisms.

We speculate that in addition to the exact impact of the antiflavivirus activity of enoxacin (far from considered a promising drug candidate), it is important to underline the potential role of an agent that is able to interfere with miRNA maturation with the aim to treat several virus infections.

A recent published article by Mori et al. described the ability of enoxacin to reduce the levels of a specific miRNA, thus extending the lifespan in *C. elegans* worm.^58^ In contrast to previous research works, here the authors highlighted the SMIR effect of enoxacin. Based on the evidence that miRNAs have been linked to aging in a wide variety of organisms,^59^ the authors used enoxacin as a proof-of-concept approach to confirm that the modulation of miRNA processing could extend the worm lifespan. At 100 μg/mL, enoxacin increased the lifespan in *C. elegans*, as measured by levels of muscular dysfunction, depending on the presence of SKN-1 protein, involved in the SKN-1/Nrf2-mitohormesis pathway. Enoxacin significantly modulated miRNA maturation, affecting the expression of 40 miRNAs (28 upregulated and 22 downregulated). However, only enoxacin-mediated miR-34-5p reduction was attributable to the observed effects in *C. elegans*, thus highlighting the SMIR behavior of enoxacin to carry out this action.

In the same article, Mori and colleagues (Figure 2, Table 1) observed that this activity was not dependent on the presence of the RDE-4, the *C. elegans* protein homologue of TRBP in humans. Thus, following an in-silico study to identify potential targets, the authors experimentally observed that enoxacin activity was directly dependent on the presence of the dsRNA-specific adenosine deaminases (ADARs) ADR-1 and ADR-2. Previously, implicated in miRNA processes and aging, these proteins can bind different classes of dsRNAs and convert adenosine to inosine.^60,61^ In this regard, Mori et al. speculated that enoxacin might stabilize the interaction between ADARs and pri-miR-34, thus preventing its processing. The authors also observed a failure of the activity of enoxacin in extending lifespan in the presence of the antioxidant *N*-acetylcysteine, suggesting that enoxacin could lead to the activation of a prooxidative/mitohormetic pathway.

## Considerations of the Mechanism of Action in the Context of Drug Discovery

The involvement of TRBP in the biological effect of enoxacin is evident in many published articles, but experiments validating a molecular binding between TRBP and enoxacin are limited to the two papers of Jin et al.^18^ and Esteller et al.^33^ (Figure 2, Table 1). Since 2011, there have been no further experiments demonstrating the binding between TRBP and enoxacin; thus, we believe that more detailed investigations should be performed to shed light on a topic that could make a change in the treatment of many human diseases.

Indeed, the recent research article of Xhemalce et al.^19^ showed PIWIL3 as the mechanistic target of enoxacin, thereby casting doubt on its correct mechanism of action. Regarding this study, some considerations should be made. A concern could be the possibility that the click chemistry step, used in the experimental procedure (see above for details), led to a false positive target because it was performed using cell lysates and, thus, in the presence of a large amount of different proteins. In addition, enoxacin showed a high IC_50_ value in different experimental models, thus suggesting a modestly high affinity to its target for chemical biology studies. Therefore, the process of cell lysis could negatively impact the pre-existing bond between the molecule and its target (commonly based on weak interactions), thus generating potential rearrangements during the experiment. Indeed, cutting-edge strategies to carry out target fishing by activity-based protein profiling (ABPP) involve the use of photoaffinity groups that are able to covalently bind the target under UV irradiation after the first interaction between the molecule and target but before cell lysis.^62^ Conversely, a possible explanation concerning why depletion of PIWIL3 in MCF7 cells showed a behavior comparable to wt MCF7 cells treated with enoxacin might involve the complex mechanism underlying piRNA expression, which is still poorly understood.^20^

The topic becomes even more complicated when considering the recent discovery that enoxacin targets ADARs in *C. elegans*. ADAR proteins are also present in humans.^63^ They have dsRBDs and are involved in the miRNA maturation pathway through their ability to bind Dicer and augment RISC loading.^63^ However, little is known about the piRNA biogenesis mechanism and ADAR1 involvement in miRNA maturation.

Therefore, there are no answers available to date regarding this enigma, raising concerns about the exact mechanism of action of enoxacin as the sole SMER reported in the literature. The ability of enoxacin to modulate/enhance miRNA maturation is well-known, but there is insufficient information to support or deny a theory regarding how enoxacin exerts its SMER activity. It must be recognized, however, that there are many clues regarding the involvement of Dicer and TRBP in the mode of action of enoxacin.

In recent years, the Dicer/TRBP complex has been extensively examined. Focusing attention on the cytoplasmatic step involving Dicer slicing, Wang et al. recently published high-resolution cryo-EM structures of the Dicer/pre-let-7 complex.^64^ In addition, attached to the DExD/H-box helicase domain of Dicer, the authors identified the third of the three dsRBDs of the cofactor TRBP.^64^ This finding explains that the DExD/H-box helicase domain serves as a platform for Dicer to bind TRBP and that its deletion indeed led to a significant decrease in the binding affinity between Dicer and pre-let-7. However, the truncated form of Dicer even improved its dicing activity, suggesting that TRBP did not take part in the catalytic activity.^64^ Indeed, as demonstrated by Joo and colleagues,^65^ TRBP ensures efficient Dicer processing in an RNA-crowded environment that does not directly contribute to the catalytic activity but rather assures accurate pre-miRNA to miRNA cutting. In the same period, Allain et al. released NMR structures of dsRBD1 and dsRBD2 of TRBP in complex with a 19-base-pair siRNA.^66^ Of note, the authors observed that RNA recognition from the dsRBDs of TRBP was completely unspecific, suggesting that TRBP possesses an unbiased binding behavior toward RNA molecules.^66^

Assuming that enoxacin binds TRBP, its function in the dicing process was questioned. Based on known information about enoxacin and its ability to enhance miRNA maturation, we would like to provide a possible explanation of the mechanism of action.

**Figure 4:**
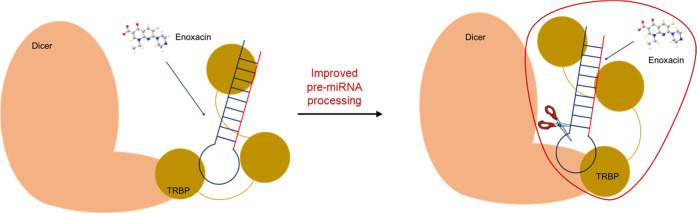
Possible interaction of enoxacin with the Dicer/TRBP/pre-miRNA complex. The red perimeter defines the area in which enoxacin may interact with the complex to improve miRNA processing.

Considering the innate ability of the quinolone class to bind (ribo)nucleic acids, a conceivable mechanism for enoxacin may involve just this feature. Indeed, on the one hand, enoxacin can bind to one of the three dsRBDs of TRBP, while on the other hand, it may strongly bind the pre-miRNA for maturation. In this manner, the role of enoxacin would be to enhance the affinity between TRBP and pre-miRNA, as experimentally reported by Jin et al.^18^ The increased affinity between TRBP and pre-miRNA would enable TRBP to rapidly load pre-miRNAs and form the Dicer/TRBP/pre-miRNA complex, thus facilitating the *in toto* dicing process (Figure 4).

This proposed mechanism would agree with the ability of enoxacin to enhance the miRNA maturation process. However, essential considerations should be addressed: how does enoxacin increase the expression of specific miRNAs without interfering with the maturation of others? More importantly, how does enoxacin specifically modulate miRNA maturation to halt the proliferation of cancer cells? In an attempt to answer these questions, enoxacin likely can bind a precise nucleotide sequence on specific pre-miRNAs, thus accounting for its preference in enhancing the maturation of some miRNAs rather than others. It would be challenging but groundbreaking to identify, using bioinformatics tools, any potential shared nucleotide sequences in regulated miRNAs to predict the pre-miRNAs that will be enhanced by enoxacin. Regarding the second question concerning how enoxacin-mediated miRNA expression regulates cell growth, an answer can be found by examining the overall increase in miRNA maturation following enoxacin treatment and considering that miRNA downregulation is often associated with cell proliferation.^16^

It is worth noting that TRBP belongs to a large family of the RBPs (such as HuR, PUM2, Musashi, LIN28, etc.), and thus, enoxacin is considered as a small molecule targeting RBP–RNA interactions. Targeting RBPs represents an attractive and challenging approach of modern drug discovery that has some strengths but also different weaknesses that are mainly related to the resulting unwanted effects.^67,68^ To the best of our knowledge, there have been no experimental findings concerning the interaction of enoxacin with other human RBPs, which, however, cannot be excluded as potential targets. In addition, the mechanism of action of enoxacin underlying the modulation of miRNA biogenesis requires an improvement of the interaction between RNA (pre-miRNA) and RBP (TRBP). Therefore, enoxacin belongs to the small molecules targeting RBP–RNA interactions but with the aim of enhancing this interaction, as experimentally demonstrated by Jin et al.^18^ Consequently, enoxacin should be placed in a different cluster with respect to the small molecules designed to inhibit the interaction with the RNA of RBPs that are often abnormally expressed in different diseases.^67,68^

At present, we are hopeful that NMR or crystallographic studies on this direction will soon be performed to confirm or refute the suggested hypothesis. Only when the exact mechanism of action at the molecular level is definitely revealed can drug discovery programs be planned in a more rational way; however, it is possible to test the many thousands of already known quinolones to identify those with an enoxacin-like mechanism of action.

## Challenges in the Development of New Anticancer Quinolone-Based SMER Compounds

Considering the large number of review articles recently published and reporting various mechanisms underlying the anticancer activity of a large number of (fluoro)quinolones,^69−72^ the scope of this section lies in discussing whether the quinolone class may be developed to obtain potent anticancer compounds that function as SMERs. In fact, in addition to the popular antimicrobial activity, the quinolone class is well-known for its anticancer effect exerted by different mechanisms; one of the most common mechanisms entails the inhibition of human topoisomerase. In this context, vosaroxin (or voreloxin) represents the quinolone-like (naphthyridone nucleus) molecule at the later stage (phase III) as an anticancer agent.^73^ Discovered in the early 2000s, as expected, its action entails the inhibition of human topoisomerase II with a dual mechanism: (*i*) stabilization of the cleavage complex topo II α/β isoforms and DNA resulting in an accumulation of DNA double-strand breaks and (*ii*) intercalation into DNA.^73^ These mechanisms ensure a certain degree of vosaroxin selectivity, such as targeting the replicating cells and being less effective against normal cells. However, vosaroxin is one of the many quinolones that act as topo II inhibitors.^69^ It appears evident that the mechanism of action of vosaroxin, like that of other quinolone topo II inhibitors, is very similar to the mechanism adopted by antimicrobial quinolones in inhibiting bacterial DNA gyrase or topoisomerase IV.^74^ This similarity in the mechanism of action, especially in the mode of action, makes the building of a differentiated SAR around the quinolone class very difficult.^75^ Indeed, both in antibacterial and anticancer activities, quinolones seem to depend mainly on the presence of the carboxylic function at the C-3 position because of the requirement for the essential Mg^2+^ ion interaction. However, after decades of efforts, it is not possible to identify substituents to add on the quinolone core to definitely obtain a specific effect. The case of vosaroxin represents a success story since it shows anticancer activity at concentrations approximately 20-fold lower than its antimicrobial effect.^73^ This distinction of activities should not be undervalued. In the early stage of a medicinal chemistry program, especially in the cancer field, the selectivity index toxicity/activity is the area of focus, thus overlooking the antibacterial effect. Then, in the later medicinal chemistry stage, once the quinolone molecule has achieved potent anticancer activity coupled with a low toxicity toward human cells, the removal of antibacterial effects becomes difficult if not impossible. Nonetheless, the antimicrobial activity must be eliminated because the long treatment required for anticancer therapy cannot be paired with an antibacterial effect. This pairing would lead to severe side effects in the patient and serious consequences for global health, uncontrollably promoting the insurgence of antimicrobial resistance.

All the issues described previously can also greatly impact the discovery of new quinolone-based SMER compounds and make their design in the context of multiple activities very interesting. Specifically, new quinolone-based SMER compounds should have a potent effect on miRNA maturation without retaining human topo II inhibition and antibacterial activity. However, in the search for a polypharmacology agent, human topo II inhibitory activity could be conserved with the aim to obtain a multitarget anticancer agent that is able to modulate miRNA maturation and poison/break DNA; toxicity against normal cells, however, should be closely monitored for any type of poisonous agent.

Returning a “clean” SMER compound, research could also be directed toward molecules with a different scaffold from the quinolone core. In this manner, reducing the antibacterial effect may be facilitated but with the loss of many of the key features of the quinolone class: (*i*) the intrinsic ability to bind RNA/DNA, (*ii*) known and ideal physicochemical properties, and (*iii*) synthetic feasibility. To date, research aimed at designing new quinolone-based SMER compounds is naive. Little is known about the SAR around enoxacin since the only available information is provided in the article published by Jin et al.^18^ However, a starting point could be based on the bonding of quinolones to Mg^2+^, which is absent in the structure of TRBP (most likely the target of enoxacin).

Support may still be derived from the article in which enoxacin was first reported as an RNAi enhancer compound.^18^ The authors defined its activity as structure and not quinolone class-dependent, thus considering enoxacin as the sole compound in the literature with this innovative mechanism of action. However, in the same article, the authors also observed that ciprofloxacin and, to a lesser extent, norfloxacin could enhance the RNAi process. In parallel, over the years, ciprofloxacin and other quinolones have often been reported as promising anticancer agents endowed with different mechanisms of action, differing from the modulation of miRNA maturation and human topo II inhibition.^69^ In our opinion, most of these compounds could be involved in modulating miRNA maturation because any interference in this process can produce several downstream changes in cellular mechanisms. We know that enoxacin is able to enhance miRNA maturation in cells; however, ignoring this effect, we can state that enoxacin can (*i*) increase p53 expression, (*ii*) improve Dicer activity, (*iii*) reduce HDAC1 and SIRT1 levels, and (*iv*) lower EMT markers, among other effects. Thus, most quinolones reported as anticancer agents with multiple mechanisms of action could serve as SMER compounds. Perhaps a step back should be taken to re-evaluate the mode of action of many anticancer quinolones to diversify those with and without SMER activity. The availability of a pool of compounds belonging to the same class would be of great relevance to build a robust SAR that may boost research on new SMER molecules.

Thus, considering the large number of quinolones present in academic groups and pharmaceutical companies, we are extremely surprised that, in more than ten years from the first publication,^18^ no other quinolone has been identified as a SMER compound. A question persists in our mind: are pharmaceutical companies working on this topic without revealing promising results or, conversely, are they failing to reveal unsuccessful data? Regardless, although challenging, research on the identification of potent SMER compounds holds promise for the identification of molecules having effects in disparate diseases. The high concentration needed to achieve SMER activity would involve the use of enoxacin at suboptimally high doses that are still suitable due to its good pharmacokinetic properties (PK) and high tolerability (highest tolerated single dose 1600 mg).^76,77^ Thus, we believe that enoxacin should be considered as the progenitor of a future class of SMER drugs, and we hope that this review will encourage the development of molecules endowed with similar PK properties and improved potency.

In this regard, considering the long-standing experience in designing and synthesizing quinolones with different biological activities,^78−83^ we undertook a wide screening to identify compounds possessing SMER activity. To date, we have collected preliminary results demonstrating that other quinolones distinct from enoxacin, already published with different activities, exhibit anticancer effects most likely acting as SMER compounds, to a greater extent than enoxacin; the results of this research will be published in the near future.

## Closing Remarks

The fluoroquinolone class of drugs, which includes the naphthyridone enoxacin, has recently received some restrictions from the EMEA and FDA due to potential permanent side effects following long-term treatment.^84,85^ However, fluoroquinolones remain an essential class of antibacterials that are commonly used to treat several infections. Due to the favorable physicochemical properties and synthetic feasibility, quinolone molecules have been extensively used in medicinal chemistry, and most of them have shown biological functions that are unrelated to their antibacterial properties.^72^

The SMER enoxacin could be considered the progenitor of a new class of drugs with different therapeutic applications: from anticancer to antiviral and from anti-ALS to antiaging effects. In addition, it is widely known that many quinolones have been reported as anticancer agents, especially as human topo II inhibitors, none of which are SMERs. In this article, we reviewed all the main activities highlighted for enoxacin, also taking into account the different targets of quinolone molecules. Although the SMER activity of enoxacin occurs at high concentrations, solid and numerous data are available regarding this innovative mode of action; thus, we feel that enoxacin may be considered a very promising template for future drug discovery programs, but the concept of polypharmacology or promiscuity should be carefully considered. The apparent promiscuity of quinolones, especially enoxacin, could be due to the SMER properties, which rely on the complexity of influencing miRNA maturation; however, the severity of some diseases (e.g., cancers, amyotrophic lateral sclerosis, and to some extent Zika infections) can justify the therapeutic use of SMERs acting on multiple miRNAs.

When this review was written, the SARS-CoV-2 pandemic struck the world. Based on the previously reported imbalance of miRNAs in coronavirus-infected cells^86−89^ and on the anti-Zika activity of enoxacin through RNAi modulation,^55^ we would like to suggest that this activity might be investigated in an attempt to counteract SARS-CoV-2 infection.

We cannot exclude the possibility that in the near future new quinolones, given their nucleic acid binding capability, will be identified and designed to discriminate between different miRNA pools. We believe that enoxacin represents a robust proof-of-concept that strongly drives away the idea of promiscuity while bringing to light future opportunities around quinolone compounds. We can define the SMER effect of enoxacin as follows: “one molecule, one pathway, multiple effects”. Thus, we vigorously support the hypothesis that enoxacin falls within the polypharmacology and will provide a platform for the next generation of drugs.

Since its discovery as a SMER compound in 2008, enoxacin has remained the only molecule reported in the literature to possess this activity. A future challenge will be to identify new SMERs, the solution to which lies in the hands of medicinal chemists.

## Abbreviation Used

ABPP: activity-based protein profiling
ADAR: dsRNA-specific adenosine deaminase
ALS: amyotrophic lateral sclerosis
ATM: Ataxia Telangiectasia Mutated
DDR: DNA-damage response
DGCR8: double-stranded RNA binding domain protein
diRNA: double-strand break-induced RNA
dsRBP: double-stranded RNA binding protein
dsRNA: double-stranded RNA
EFST: Ewing sarcoma family tumors
EGFP: enhanced green fluorescent protein
EMA: European Medicines Agency
EMT: epithelial-mesenchymal transition
EVs: extracellular vesicles
HEK293: human embryonic kidney
hNPCs: human neural progenitor cells
HR: homologous recombination
iPSCs: induced pluripotent stem cells
LNAs: locked nucleic acids
miRNA: microRNA
NHEJ: nonhomologous end joining
piRNA: Piwi-interacting RNA
RISC: RNA-induced silencing complex
RNAi: RNA-interference
SAR: structure–activity relationship
SGs: stress granules
shRNA: short-hairpin RNA
siRNA: short-interfering RNA
SMER: small-molecule enhancer of microRNA
SMIR: small molecule inhibitors of specific miRNA
SPR: surface plasmon resonance
TDP-43: TAR DNA-binding protein 43
TRBP: TAR-RNA binding protein
UTR: untranslated region
vsiRNAs: virus-derived small interfering RNA
ZIKV: Zika virus
